# Matter-wave interference of a native polypeptide

**DOI:** 10.1038/s41467-020-15280-2

**Published:** 2020-03-19

**Authors:** A. Shayeghi, P. Rieser, G. Richter, U. Sezer, J. H. Rodewald, P. Geyer, T. J. Martinez, M. Arndt

**Affiliations:** 1 Faculty of Physics, University of Vienna, VCQ, Boltzmanngasse 5, A-1090 Vienna, Austria; 2 Centre for Cold Matter, Blackett Laboratory, Imperial College London, Prince Consort Road, London, SW7 2AZ United Kingdom; 3 Department of Chemistry and the PULSE Institute, Stanford University, Stanford, CA 94305 USA; 4 SLAC National Accelerator Laboratory, Menlo Park, CA 94025 USA

**Keywords:** Atomic and molecular interactions with photons, Matter waves and particle beams, Mass spectrometry

## Abstract

The de Broglie wave nature of matter is a paradigmatic example of quantum physics and it has been exploited in precision measurements of forces and fundamental constants. However, matter-wave interferometry has remained an outstanding challenge for natural polypeptides, building blocks of life, which are fragile and difficult to handle. Here, we demonstrate the wave nature of gramicidin, a natural antibiotic composed of 15 amino acids. Its center of mass is delocalized over more than 20 times the molecular size in our time-domain Talbot-Lau interferometer. We compare the observed interference fringes with a model that includes both a rigorous treatment of the peptide’s quantum wave nature as well as a quantum chemical assessment of its optical properties to distinguish our result from classical predictions. The realization of quantum optics with this prototypical biomolecule paves the way for quantum-assisted measurements on a large class of biologically relevant molecules.

## Abstract (web summary)

Matter-wave interferometry of complex molecules is challenging due to difficulties in preparing and detecting molecular beams. Here the authors demonstrate quantum behavior of a polypeptide using matter-wave interference in an all-optical time-domain Talbot-Lau interferometer.

## Introduction

The wave nature of massive particles is a central aspect of quantum physics. The free evolution of particles is no longer described by classical trajectories, but instead by a wave-like propagation in multiple directions. Recombining the wavefronts leads to interference, where the probability amplitude for a particle arriving at a certain position depends on the phase difference of the partial waves. Since these phases are sensitive to even small perturbations, matter-wave interferometry has become an important tool for atom optics^1,2^, probing fundamental physics^3–7^ or serving in advanced quantum sensors^8–10^. The de Broglie wave nature has also been shown for large molecules, from fullerenes^11^ and molecular clusters^12^ up to tailor-made macromolecules^13^. Such experiments probe the quantum-to-classical interface and can be used to characterize neutral molecules in the gas phase, through interference fringe deflection in electric and magnetic fields^14^ or minimally invasive spectroscopy^15,16^.

Until today, quantum optics with fragile natural biomolecules has remained elusive due to the challenges in forming stable and intense molecular beams which can be detected with high efficiency and selectivity. Measurements on neutral biomolecules in the gas phase will, however, become valuable as they are solvent-free and allow predicting and evaluating their electronic properties independent of any matrix environments^17^. A typical matter-wave experiment requires an efficient source to launch neutral particles in high vacuum, beam splitters to coherently prepare, separate and recombine the quantum wave function associated with the molecular center-of-mass motion and an efficient detector with high sensitivity and mass resolution to record the result. For atom interferometry, these challenges have already been elegantly solved^2^. For interferometry with complex biomolecules, sources are a prime challenge. While evaporation and sublimation can still be used for vitamins and tripeptides^18,19^, it denatures and decomposes more complex polypeptides. And while matrix assisted laser desorption^20^ and electrospray ionization^21^ can volatilize even large proteins, they produce ions which are prone to dephasing and decoherence in quantum experiments. Direct laser desorption using nanosecond laser pulses has proven useful to entrain neutral peptides into cold noble gas jets where selected species could be detected using photoionization with VUV radiation^22^. However, energetic nanosecond pulses typically ablate large amounts of clusters and nanoparticles^23^ in addition to the individual peptides that are desired.

In the following, we present a realization of matter-wave interferometry of *gramicidin A1*, a linear antibiotic polypeptide composed of 15 amino acids with a mass *m* = 1882 amu = 3.13 × 10^−24^ kg. It has many desirable properties for such an experiment. And as a natural amino acid sequence, produced by the soil bacterium *Bacillus brevis*, it is representative for a large class of biologically relevant molecules. In addition, it contains four tryptophan residues, which is the only one of all 20 natural amino acids that is ionizable with a single vacuum ultraviolet (VUV) photon with energy 7.9 eV. This is crucial for diffraction and detection of the neutral peptides^24^.

## Results

### Experiment

**Fig. 1:**
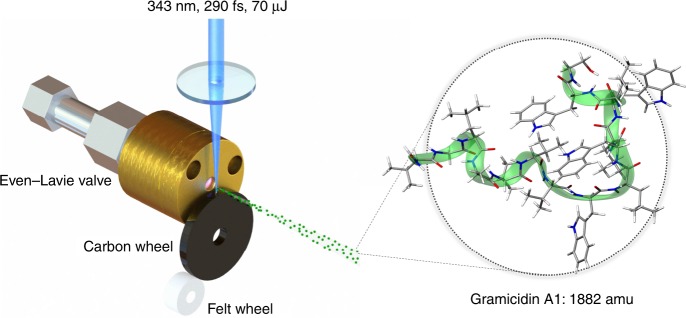
Peptide source: Ultra-fast 290 fs laser pulses with an energy of up to 70 μJ and a wavelength of 343 nm are focused to a spot diameter of 100 μm to desorb gramicidin molecules from a glassy carbon wheel. The molecules are picked up by an adiabatically expanding argon (helium) jet at 600 ms^−1^ (1200 ms^−1^) from a short-pulse high-pressure valve. The emerging polypeptide matter-wave has a de Broglie wavelength of 350 fm (175 fm). Gamicidin A1 is a 15 amino acid polypeptide. The green ribbon runs along the peptide bonds and the residues are shown as line diagrams. The four Tryptophan residues are the important chromophores that enable pulsed VUV laser ionization and thus the realization of optical diffraction gratings and photo-ionization in combination with mass-sensitive detection in our matter-wave interferometer. Parts of this figure have been adapted from reference^24^.

We use the idea presented in Fig. 1: A rotating carbon wheel coated with a biomolecular film serves as the sample supply. From there, the molecules are desorbed by pulsed laser light and entrained in a supersonically expanding noble gas jet. While nanosecond lasers are known to deliver intact peptide beams^22,25^, ultrafast laser pulses with TW/cm^2^ intensities and pulse lengths of  290 fs allow for 30-fold improvement in sample efficiency^24^. The gramicidin beam is then skimmed, vertically and horizontally collimated to <1 mrad, and sent into the interferometer chamber. In our setup we obtain a velocity *v* = 600 ms^−1^ when using argon and *v* = 1200 ms^−1^ when using helium as a carrier gas. The different velocities are used to access different de Broglie wavelengths *λ*_dB_ = *h*∕*mv*, where *h* is Planck’s constant.

**Fig. 2:**
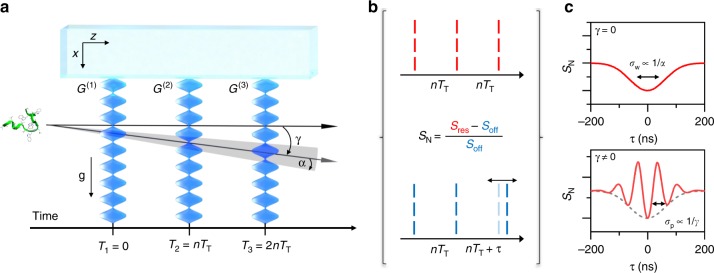
Time-domain matter-wave interferometry: Matter-wave interferometer (**a**): Three retro-reflected VUV laser beams realize the standing light waves as pulsed photo-depletion gratings. The antinodes in *G*^(1)^ prepare a comb of tightly confined positions from where a molecule may emerge. Because of this projective confinement the wave coherence rapidly expands in free flight to cover several nodes and antinodes by the time the second grating fires. Rephasing of the matter-wave behind *G*^(2)^ then leads to de Broglie interference of each molecule with itself and to the formation of a periodic molecular density pattern around the time when *G*^(3)^ is fired. Only molecules whose wave functions are aligned with the nodes of *G*^(3)^ are transmitted to the detector. The coherent rephasing occurs around a characteristic timescale, the *n*-th multiple of the Talbot time. A typical measurement (**b**): we toggle between two interferometer modes: a symmetric mode (resonance), where the grating pulse separation times are kept equal and close to *nT*_T_, and an asymmetric mode (off-resonant or reference), where we set an imbalance of up to 200 ns. Imprinted fringes (**c**): If the molecular beam velocity has a component parallel to *x*, the fringe pattern effectively has a transverse velocity component and its position relative to the third grating becomes time dependent. A fringe pattern is visible in case the divergence angle *α* is smaller than the tilt angle *γ*.

We send the peptides through our time-domain Talbot-Lau interferometer^12^, where three pulsed VUV light gratings *G*^(1)^ − *G*^(3)^ ionize and remove the molecules in the antinodes of the laser fields (see Fig. 2). The gramicidin molecules arrive with a de Broglie wavelength of *λ*_dB _= 350 fm (at *v* = 600 ms^−1^) which is about 10^4^ times smaller than the molecular size. We select a velocity spread of *Δv*∕*v* ≃ 0.5%, defined by the duration of the carrier gas pulse (20 *μ*s) and the beam width (3 × 3 mm^2^) of the detection laser. This corresponds to a longitudinal (spectral) de Broglie coherence of ca. 200*λ*_dB_ ≃ 72 pm. Equally important is the transverse (spatial) coherence which sets an upper limit to the useful width of diffractive elements, across which matter-wave phenomena are relevant. Upon arrival at *G*^(1)^, it is of the order of 200 pm, too small for diffraction at *d* = 78.8 nm gratings. However, by defining a precise starting position of the molecules, one can increase their quantum mechanical momentum uncertainty, thus boosting transverse coherence further downstream such that the coherence function covers several effective slits in the second grating. The initial confinement in *G*^(1)^, diffraction in *G*^(2)^ and position sensing in *G*^(3)^ is done by a position measurement: the molecules can only pass to the detector if they fly through the nodes of all three VUV grating. *G*^(1)^ thus prepares the required coherence, *G*^(2)^ diffracts the matter-wave and interference results in a molecular density pattern that is modulated by *G*^(3)^ with nanoscale spatial sensitivity.

The gratings *G*^(1)^ − *G*^(3)^ are formed by reflecting three fluorine (F_2_ excimer, *λ*_L _= 157.63 nm) laser beams from a single dielectric mirror. This makes the interferometer robust against vibrational dephasing but it also impedes scanning of *G*^(3)^ across the density pattern. However, a tilt *γ* of the molecular beam relative to the mirror surfaces allows us to scan the matter-wave fringes across *G*^(3)^ by varying the pulse delay between *G*^(2)^ and *G*^(3)^^12,26^, as seen in Fig. 2b). For that purpose, we vary the pulse separation time *T* between the second and third grating in two complementary settings. In the resonant mode, *T*_3_ − *T*_2_ = *T*_2_ − *T*_1_ are set to the *n*-th multiple of the Talbot time

1 $${T}_{{\rm{T}}}=\frac{m{d}^{2}}{h},$$

with the grating period *d* = *λ*_L_∕2. Then, the near-field resonance condition is met and a clear interference pattern appears. The transmitted molecular signal ${S}_{{\rm{res}}}$ through *G*^(3)^ depends on the position of the grating nodes relative to the matter-wave fringes. In the off-resonant (reference) mode, the signal *S*_off_ is recorded while *G*^(3)^ is shifted by a variable time ∣*τ*∣ ≤ 200 ns. For our Talbot time and beam divergence *α*, the 200 ns shift is sufficient to smear out the interference pattern. The interference contrast is then defined as the normalized signal difference ${S}_{{\rm{N}}}=({S}_{{\rm{res}}}-{S}_{{\rm{off}}})/{S}_{{\rm{off}}}$^12^ (Fig. 2c).

### Modeling

We model the expected signal by evolving the transverse Wigner function *w*(*x*, *p*_*x*_)^27^, taking into account tilted and divergent molecular beams, as well as mirror and grating imperfections^28^. As a phase space description, it allows comparing the experiment with both the quantum and the classical expectation within the same framework (see Methods). The free evolution of a particle with initial position *x* and momentum *p*_*x*_ is then described by a shearing transformation $w\left(x,{p}_{x}\right)\to w\left(x-{p}_{x}t/m,{p}_{x}\right)$. Additional terms are introduced to account for earth’s gravitational acceleration *g* and a tilt of the molecular beam by an angle *γ* with respect to the mirror surface, which results in an additional constant transverse momentum ${p}_{\gamma }=mv\tan (\gamma )$. The signal seen by the detector depends on the Talbot time *T*_T_, the pulse delay *τ* of *G*^(3)^ with respect to the Talbot time and the relative shift of the grating nodes to the fringe pattern. Each grating modulates the molecular wave function both in its amplitude and phase via photon absorption and the optical dipole potential, respectively. The transmission function of the *k*-th grating

2 $${t}^{(k)}(x)=\exp \left[\left(-\frac{{n}_{0}^{(k)}}{2}+i{\phi }_{0}^{(k)}\right){\cos }^{2}\left(\frac{\pi x}{d}\right)\right]$$

depends on the number of absorbed photons ${n}_{0}^{({\rm{k}})}$ and the acquired phase shift ${\phi }_{0}^{({\rm{k}})}$ at an antinode, which both contribute to the dimensionless parameter *β*^27^

3 $$\beta =\frac{{n}_{0}^{({\rm{k}})}}{2{\phi }_{0}^{({\rm{k}})}}=\frac{{\lambda }_{{\rm{L}}}}{8{\pi }^{2}}\frac{\sigma ({\lambda }_{{\rm{L}}})}{{\alpha }_{V}({\lambda }_{{\rm{L}}})}.$$

It describes the ratio of the molecule’s wavelength-dependent absorption coefficient *σ*(*λ*_L_) and its optical polarizability volume *α*_*V*_(*λ*_L_) (converted to the polarizability in SI units via *α*[SI] = 4*πε*_0_ × *α*_*V*_(*λ*_L_)). The absorption cross section determines the ionization probability and thus controls the effective slit width in all three gratings. The optical polarizability determines the phase the gramicidin molecules acquire during their transit through *G*^(2)^. It leaves the fringe periodicity unchanged but modulates the contrast. Since the molecules enter in a variety of different vibrational, rotational and conformational states, as well as orientations, the measured fringes represent an average over the internal properties.

An interferometer with three absorptive gratings would allow for the creation of classical Moiré-like patterns. This assumes molecules to be particles following ballistic trajectories that are modified by the gradient forces arising from the interaction between the optical dipole potential of the grating and the molecule’s optical polarizability. The difference between the quantum and the classical expectations is encoded in how these optical properties enter the transmission function (see Methods). The behavior of the fringe contrast is governed by *β*, making this a crucial parameter for a quantitative distinction between classical and quantum effects. A thorough understanding of the final signal therefore requires knowledge about the electronic properties of gramicidin with respect to its ground and excited states. This is a challenge since gramicidin has many possible conformational states and one has to evaluate electronic properties for an ensemble populating a complex potential energy surface (PES). The gramicidin molecule contains 1010 electrons which renders electronic structure calculations demanding even without global optimization of the conformational space and when combined with density functional theory (DFT). Here, we perform short ab-initio molecular dynamics (AIMD) simulations at 300 K, assuming the worst case of no internal cooling, to explore the conformational PES and get a measure of the dynamic polarizability volume of gramicidin (see Methods). Molecular geometries are extracted from the AIMD simulation every picosecond and are fed into subsequent DFT calculations to estimate the ensemble average of the optical polarizability volume ${\left\langle {\alpha }_{V}({\lambda }_{{\rm{L}}})\right\rangle }_{300{\rm{K}}}$.

In addition, the absorption cross section has to be determined as a thermal average ${\left\langle \sigma ({\lambda }_{{\rm{L}}})\right\rangle }_{300{\rm{K}}}$ for the calculation of *β*. The relevant relaxation channels after photon absorption are ionization and disscociation, since our detector is only sensitive to the depletion of the molecular beam: *σ* = *σ*_PI_ + *σ*_PD_, where *σ*_PI_ and *σ*_PD_ are the photoionization and the photodissociation cross sections, respectively. A lack of detected fragments indicates a comparatively small *σ*_PD_, making *σ*_PI_ a strong lower bound for the total cross section. It is measured under identical conditions in an independent experiment by monitoring the gramicidin ion count rate

4 $${N}_{{\rm{I}}}={N}_{{\rm{0}}}(1-{e}^{-{\sigma }_{{\rm{PI}}}\phi }),$$

as a function of the VUV photon fluence *ϕ*.

**Fig. 3:**
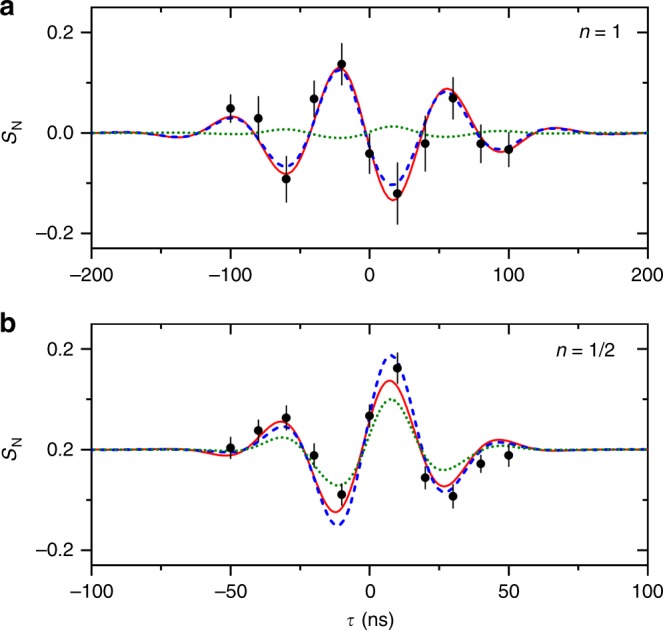
Molecular interference patterns of gramicidin: Experimental data is presented for the first (**a**) and half (**b**) Talbot order (black circles) including 1*σ* error bars. A fit according to Eq. (5) is shown (solid red line) together with a quantum-(dashed blue line) and a classical predicition (dotted green line). The fringes appear on the time-domain resonance dip when the pulse separation time between *G*^(2)^ and *G*^(3)^ is varied by a small delay *τ* around the Talbot resonance for the case of a tilted molecular beam. The envelope of the resoance dip is determined by the molecular divergence angle while the fringe period is determined by the tilt angle with respect to the mirror surface. Note the different scaling of the abscissa in **a** and **b** and that both interference orders are *d*-periodic.

These tools at hand, we can now analyze the matter-wave interferogram obtained with gramicidin, both in the first (*n* = 1) and fractional (*n* = 1∕2) Talbot order. We record them by shifting the third grating around the ’resonant’ interference mode in steps of 20 ns (*n* = 1) and 10 ns (*n* = 1/2) at a fixed time delay for the off-resonant reference signal at *τ*_off_ = 200 ns (*n* = 1) and 100 ns (*n* = 1/2). For a finite divergence and tilt of the molecular beam, the fringe density pattern scans across the grating when *τ* is varied. We expect a sinusoidal modulation with a Gaussian envelope^26^ (see Figs. 2c and 3).

5 $${S}_{{\rm{N}}}={V}_{0}\exp \left[-{\left(\frac{\tau }{{\sigma }_{{\rm{w}}}\sqrt{2}}\right)}^{2}\right]\cos \left(2\pi \frac{(\tau -{\tau }_{{\rm{off}}})}{{\sigma }_{{\rm{p}}}}\right).$$

The modulation of the fringe visibility *V*_0_ allows us to more precisely determine the divergence angle *α* = 0.4 mrad from the width *σ*_w_ of the resonance dip

6 $$\alpha =\arcsin \left(\frac{d}{2v{\sigma }_{{\rm{w}}}\sqrt{2 \, \mathrm{ln}\,10}}\right),$$

and the tilt angle *γ* = 1.7 mrad from the observed fringe period *σ*_p_

7 $$\gamma =\arcsin \left(\frac{d}{v{\sigma }_{{\rm{p}}}}\right).$$

We extract the model parameters *α* and *γ* from the data in Fig. 3, the absorption cross section from independent measurements and the VUV polarizability volume from our quantum chemical analysis.

The dynamic polarizability volume is computed by Q-Chem^29^ using DFT with the range-separated hybrid exchange-correlation functional LC-*ω*PBEh^30^, which has been shown to perform well for the calculation of polarizabilites^31^, and the 6-31G basis set. The Coupled-Perturbed Kohn-Sham method^32^ is used to calculate the optical polarizability volume for every extracted geometry at *λ*_L_ to obtain the ensemble average ${\left\langle {\alpha }_{V}({\lambda }_{{\rm{L}}})\right\rangle }_{300{\rm{K}}}$ = (157 ± 1) × 10^−30 ^m^3^.

**Fig. 4:**
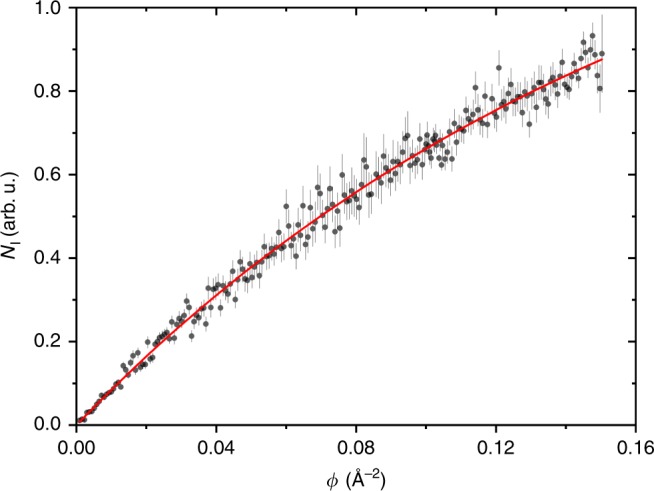
Ion counts as a function of the photon fluence *ϕ*: The error bars represent the standard deviation of the count rate, assuming Poissonian statistics. The ionization cross section *σ*(*λ*_L_) can be extracted according to Eq. (5). The single exponential increase in the range of available laser intensities is compatible with a single-photon process.

In order to obtain the photoionization cross section *σ*_PI_, we measure and plot the number of ions *N*_I_ as a function of the photon fluence *ϕ* (see Methods, Fig. 4) and construct a fit according to Eq. (4). We find an ionization cross section of *σ*_PI_ = ${\left\langle \sigma ({\lambda }_{{\rm{L}}})\right\rangle }_{300{\rm{K}}}$ = (4.7 ± 0.2) × 10^−20 ^m^2^ and thus *β* ≃ 0.6. This value is used for simulating both the quantum and classical predictions.

The final result is shown in Fig. 3, which compares the experimental data (black circles), with a fit based on Eq. (5) (solid red line), the quantum simulation (dashed blue line) and the classical description (dotted green line). Figure 3a presents data for the first Talbot order, while Fig. 3b shows the *n* = 1/2 Talbot order, where the peptides are entrained in helium to double their mean velocity to 1200 ms^−1^. In both cases, the resulting molecular density pattern at *G*^(3)^ has a fringe separation of *d* = 78.8 nm since at the half Talbot order (*n* = 1∕2), the halving of the fringe period in time is caused by the doubling of the molecular velocity.

In the first Talbot order, the experimental fringe contrast is very well described using quantum wave mechanics (blue dashed line) while a description using classical trajectories (green dotted line) misses the amplitude by almost an order of magnitude. On the other hand, both models approach each other and the experiment at the half Talbot order (Fig. 3b). At very short intervals between the gratings, there is too little time for the molecular matter-waves to spread out in space.

Claiming the quantum nature of the fringe pattern requires to check if there is any reasonable way to reproduce the observed fringe contrast at *n* = 1 in a classical model, for instance assuming molecular properties that differ from their computed or measured values. We find that *β* ≃ 100 rather than *β* ≃ 0.6 would be required for the classical curve to mimic the quantum result. This is vastly incompatible with the calculations and observations described above and also inconsistent with *β* values found in many other organic molecules and clusters^28^. The quantum model appears to be the only plausible explanation for our experimental results.

One may also ask for the role of external forces, such as gravity, in this setting. The normalized signal difference is a function of the pulse separation time *T* and the Earth’s gravitational acceleration *g* via ${S}_{{\rm{N}}}(T)={V}_{0}\sin \left(2\pi (b-g{T}^{2})/d\right)$, with *b* a constant offset on the laser mirror^26^. At fixed Talbot order, gravity influences the absolute fringe height, but within the 100 ns fringe envelope, of Fig. 3 gravity does not cause any noticeable time-dependence.

In summary, we have demonstrated matter-wave interferometry with a complex native polypeptide, the antibiotic gramicidin. The fringe visibility of around 20% in the first Talbot order stands in marked discrepancy to a classical phase space description and is in very good agreement with quantum mechanics, including a detailed quantum chemical analysis of the molecular electronic properties. Our source techniques based on UV femtosecond desorption can volatilize fragile biomolecules in a more efficient way than other methods to date. While matter-wave experiments with biomolecules in the gas phase do not elucidate biological function per se—which is related to electronic structure determining folding dynamics and interactions with matrix environments—our experiments show that quantum phenomena can be observed with building blocks of life under suitable boundary conditions. Molecular interference patterns can be used as flying nanorulers^33^ that will become important in studies of optoelectronic and structural properties of complex biomolecules.

## Methods

### Sample preparation

Gramicidin D (Sigma Aldrich, CAS: 1405-97-6) is used which is a mixture of different antibiotic compounds. The major component is gramicidin A1, a linear polypeptide composed of 15 amino acids. It has the chemical formula C_99_H_142_N_20_O_17_. The molecule sketched in Fig. 1 represents one specific configuration of gramicidin A1. The green ribbon follows the peptide sequence while the tryptophan, valine and isoleucine rest groups are explicitly shown. The source emits a large variety of structural conformers, which all contribute to the same matter-wave interference pattern, since their mass and VUV optical properties are nearly identical. There are both molecular fermions and bosons in the sample, but quantum statistics is irrelevant in our single-molecule interference experiments. All molecules are excited in several of their 828 vibrational modes and highly excited in their rotational degrees of freedom.

### Molecular beam

The experiment runs at 100 Hz. In every cycle, an Even-Lavie valve releases a 20 μs short and dense pulse of argon with a backing pressure of about 30 bar. A femtosecond laser (Topag PHAROS, 290 fs, 70 μJ, 343 nm) is focused ($\varnothing$ = 100 μm) onto the surface of a glassy carbon wheel coated with gramicidin to create a cloud of isolated molecules. The argon (helium) pulses then entrain the molecules with a mean velocity of around 600 ms^−1^ (1200 ms^−1^). Further downstream the particle beam is skimmed (Beam Dynamics skimmer, $\varnothing$ = 2 mm), collimated to a rectangular shape of 0.6 × 1 mm^2^ (the longer axis parallel to the grating vectors) and finally transferred to the interferometer chamber via a differentially pumped stage. The pressure in the main chamber is 2 × 10^−9^ mbar, in the source chamber 1 × 10^−8^ mbar. Molecular beam velocities are determined by comparing the timing of the desorption laser with the detection laser pulse.

### Grating lasers

The grating laser beams are emitted by three GAM EX50 fluorine lasers (*λ*_L_ = 157.6 nm, 4 mJ, 8 ns, flat top profile). All beams are reflected by the same dielectric mirror 3 × 5 cm^2^, coated onto a 2 cm thick CaF_2_ substrate with the best technically available reflectivity in this wavelength range to date, i.e., *R* ≃ 97%. The laser waists are elongated parallel to the molecular beam axis *z* (10 × 1 mm^2^) and spatially separated by  ≈2 cm which allows molecules of different velocities to interact with a laser grating at the same time.

### Mirror imperfections

For a perfectly flat mirror and in the absence of external accelerations, *S*_N_ is positive and equal to the theoretical visibility. In a real world scenario, *S*_N_ is given by the visibility of the molecular density pattern at the position of *G*^(3)^ and its relative displacement *ΔD* = *Δx*_1_ −2*Δx*_2_ + *Δx*_3_ of this Talbot image with respect to *G*^(3)^. Here, *Δx*_*i*_ captures both the possible mirror corrugations at either grating site or the displacement of the molecular fringe due to external accelerations—for instance gravity. The molecular transmission is maximized for *ΔD* = *nd* and minimized for *ΔD* = (*n* + 1∕2)*d* with $n\in {\mathbb{Z}}$. If the mirror surface had deformations exceeding 10 nm across the 10 mm grating laser beam profile, molecules of the same gas pulse but with different velocities would experience differently shifted interferometers. To avoid the ensuing reduction in fringe contrast, the effective grating width is set to  <3 mm by the geometry of the final detection laser.

### Vibrational stability

The most important vibration frequencies in the experiment are related to the frequencies of the turbomolecular pumps. Taking 3000 Hz as a higher order worst case scenario the amplitude required for a 1∕*e* contrast reduction is  ≈15 nm, while the noise at that frequency is estimated to be  ≈1 nm.

### Coriolis force

Considering the latitude (48.22^∘^N) and orientation (162^∘^SSE) of the molecular beam on Earth and assuming a velocity of 600 ms^−1^ the Coriolis force on gramicidin causes a shift of around 1 nm. Even for large velocity spreads phase averaging can be neglected. Also path-length differences arising from the Coriolis force are negligible for the mass considered here.

### Optical polarizability volume at *λ*_L_

The AIMD (BLYP^34,35^/6-31G) simulations are performed using the TeraChem program package^36,37^. During the AIMD run, a single molecule is propagated over 50 ps in time steps of 1 fs at a temperature of 300 K, which is controlled by a Bussi-Parinello thermostat^38^ with a relaxation time of 0.1 ps. The dynamic polarizability volume is computed by Q-Chem^29^ using DFT with the range-separated hybrid exchange-correlation functional LC-*ω*PBEh^30^ and the 6-31G basis set. The Coupled-Perturbed Kohn-Sham method^32^ is used to calculate the optical polarizability for every extracted geometry at *λ*_L_ to obtain the ensemble average. LC-*ω*PBEh has been shown to perform well for the calculation of polarizabilites^39^.

### Ionization cross section at *λ*_L_

In order to obtain *σ*_PI_, we measure and plot the number of counted ions *N*_I_ as a function of the photon fluence *ϕ* (see Fig. 4), which is the total number of photons per unit area integrated over the laser pulse length:

8 $${N}_{{\rm{I}}}={N}_{{\rm{0}}}(1-{e}^{-{\sigma }_{{\rm{PI}}}\phi }),$$

where *σ*_PI_ and the total number of molecules *N*_0_ enter as fit parameters.

### Data analysis

In order to calculate *S*_N_, mass spectra are summed up and subtracted from the background in both measurement modes to obtain ${S}_{{\rm{res}}}$ and ${S}_{{\rm{off}}}$. There is a systematic error by assuming that the mass signals are proportional to the number of detected molecules. We thus consider a worst case scenario where every event at the detector is attributed to a single detected molecule *N*_event_ = 1. We then compare the amplitudes of our mass signals within a threshold value, that is derived from the standard deviation of the background noise. The probability of not detecting a particle *P*_zero_ is assumed to follow Poissonian statistics

9 $${P}_{{\rm{zero}}}=\frac{{N}_{{\rm{event}}}}{{N}_{{\rm{frames}}}}={{\rm{e}}}^{-{\lambda }_{{\rm{P}}}}.$$

with *λ*_P_ as the average number of counts per frame. The total number of detected molecules *N* within one measurement consisting of *N*_frames_ is then given by

10 $$N={N}_{{\rm{frames}}}(-\mathrm{ln}\,{P}_{{\rm{zero}}}),$$

while Gaussian error propagation delivers the 1*σ* error-bars of each data point.

### Quantum model of the interference fringes

Our beam experiments are supported by phase space simulations as introduced by Nimmrichter and Hornberger for near-field matter-wave interferometry^40^ and refined for time-domain experiments^27^. We here adapt the model to the details of our study. The simulations are based on the one-dimensional Wigner function *w*(*x*, *p*_*x*_) with *x* and *p*_*x*_ for the positions and the momenta of states, respectively. The Wigner function is defined as the transformation of the position density matrix $\rho (x,x^{\prime} )=\left\langle x\right|\hat{\rho }\left|x^{\prime} \right\rangle$^41^

11 $$w\left(x,{p}_{x}\right)=\frac{1}{2\pi \hslash }\int \ ds\ {e}^{i{p}_{x}s/\hslash }\left\langle x-\frac{s}{2}\right|\hat{\rho }\left|x+\frac{s}{2}\right\rangle ,$$

where the molecular beam propagation at a time *t* is represented by the Hamiltonian ${{\mathcal{H}}}_{0}={p}_{x}^{2}/2m$ in absence of external fields. The Wigner function therefore transforms like

12 $$w\left(x,{p}_{x}\right)\to w\left(x-\frac{{p}_{x}\ t}{m},{p}_{x}\right).$$

The formalism allows a simple comparison with classical phase space dynamics based on ballistic trajectories. Under free evolution the classical phase space density transforms like the Wigner function^42^.

Position shifts due to constant accelerations parallel to the grating vectors (*x*-axis) can be included to account for gravitational, electric or magnetic forces. Additionally, a tilt of the molecular beam by the angle *γ* can be introduced as a constant momentum ${p}_{\gamma }=mv\tan (\gamma )$ parallel to the *x*-axis. The Wigner function for free propagation over a time *t* therefore reads

13 $$w\left(x,{p}_{x}\right)\to w\left(x-\frac{{p}_{x}t}{m}+\frac{{p}_{\gamma }t}{m}+\frac{g{t}^{2}}{2},{p}_{x}-{p}_{\gamma }-amt\right),$$

Transmission through the *k*-th grating *G*^(k)^ is described by a complex transmission function *t*^(k)^(*x*) acting on the position density matrix

14 $$\rho (x,x^{\prime} )\to {t}^{({\rm{k}})}(x)\rho (x,x^{\prime} ){t}^{({\rm{k}})}{(x)}^{* }$$

while ${\left|{t}^{({\rm{k}})}(x)\right|}^{2}$ gives the probability for a particle at position *x* to remain in the beam and is assumed to follow poissonian statistics

15 $${\left|{t}^{({\rm{k}})}(x)\right|}^{2}=\exp \left(-{n}^{({\rm{k}})}(x)\right),$$

where *n*^(k)^(*x*) is the number of absorbed photons and shows a *d*-periodic modulation

16 $${n}^{({\rm{k}})}(x)={n}_{0}^{({\rm{k}})}{\cos }^{2}\left(\frac{\pi x}{d}\right).$$

While ${\left|{t}^{({\rm{k}})}(x)\right|}^{2}$ describes a pure absorptive grating, the additional phase modulation *ϕ*^(k)^(*x*) is described by

17 $${\phi }^{({\rm{k}})}(x)={\phi }_{0}^{({\rm{k}})}{\cos }^{2}\left(\frac{\pi x}{d}\right).$$

Here ${n}_{0}^{({\rm{k}})}$ is the average number of absorbed photons in an antinode and ${\phi }_{0}^{({\rm{k}})}$ the eikonal phase, gained by integration of the interaction potential over the intensity profile of the laser. For the optical gratings they read^43^

18 $${n}_{0}^{({\rm{k}})}=\frac{4\sigma ({\lambda }_{{\rm{L}}}){E}^{({\rm{k}})}{\lambda }_{{\rm{L}}}}{hc{A}_{{\rm{L}}}}, \quad {\phi }_{0}^{({\rm{k}})}=\frac{16{\pi }^{2}{E}^{({\rm{k}})}{\alpha }_{V}({\lambda }_{{\rm{L}}})}{hc{A}_{{\rm{L}}}},$$

where *E*^(k)^ is the pulse energy, *A*_L_ the illuminated area, *c* the speed of light, *σ*(*λ*_L_) the absorption cross section and *α*_*V*_(*λ*_L_) the optical polarizability at the grating wavelength *λ*_L_. Taking imperfections into account such as a mirror reflectivity *R* = 0.97 and a grating coherence factor *C* = 0.76^28^, there is an effective reduction of the coherent contribution to ${n}_{0}^{({\rm{k}})}$ and ${\phi }_{0}^{({\rm{k}})}$

19 $${n}_{0,{\rm{eff}}}^{({\rm{k}})}=RC{n}_{0}^{({\rm{k}})}, \quad {\phi }_{0,{\rm{eff}}}^{({\rm{k}})}=RC{\phi }_{0}^{({\rm{k}})}.$$

Using these parameters the complex transmission function of the optical gratings reads

20 $${t}^{({\rm{k}})}(x)=\exp \left(-\frac{{n}_{{\rm{eff}}}^{({\rm{k}})}(x)(1+R)}{4RC}+i{\phi }_{{\rm{eff}}}^{({\rm{k}})}(x)\right).$$

Transmission through a grating is described by the convolution of the Wigner function and the transmission kernel

21 $$w\left(x,{p}_{x}\right)\to \int d{p}_{0}{T}^{({\rm{k}})}\left(x,{p}_{x}-{p}_{0}\right)w(x,{p}_{0}).$$

For optical gratings the transmission kernel *T*^(k)^(*x*, *p*) consists of the Talbot coefficients ${B}_{{\rm{n}}}^{({\rm{k}})}(\chi )$, gained by Fourier expansion of the transmission function ${t}^{({\rm{k}})}\left(x\right)$

22 $${T}^{({\rm{k}})}\left(x,{p}_{x}\right)=\frac{1}{2\pi \hslash }\sum _{n}\exp \left(\frac{2\pi inx}{d}\right) \times {\int} ds\ {e}^{i{p}_{x}s/\hslash }{B}_{n}^{({\rm{k}})}\left(\frac{s}{d}\right),$$

23 $${B}_{n}^{({\rm{k}})}(\chi )= 	\exp \left(\frac{-{n}_{0,{\rm{eff}}}^{({\rm{k}})}}{2}\right){\left(\frac{\sin \left(\pi \chi \right)-\beta \cos \left(\pi \chi \right)}{\sin \left(\pi \chi \right)+\beta \cos \left(\pi \chi \right)}\right)}^{\frac{n}{2}}\\ 	\times {J}_{n}\left({\rm{sign}}\left(\frac{\sin \left(\pi \chi \right)}{\beta }+\cos \left(\pi \chi \right)\right)\frac{{n}_{0,{\rm{eff}}}^{({\rm{k}})}}{2\beta }\sqrt{{\sin }^{2}\left(\pi \chi \right)-{\beta }^{2}{\cos }^{2}\left(\pi \chi \right)}\right),$$

with the dimensionless parameter *β* as the ratio of molecular absorption cross section and optical polarizability containing information about the electronic structure of the considered molecules

24 $$\beta =\frac{{n}_{0}^{({\rm{k}})}}{2{\phi }_{0}^{({\rm{k}})}}=\frac{{\lambda }_{{\rm{L}}}}{8{\pi }^{2}}\frac{\sigma ({\lambda }_{{\rm{L}}})}{{\alpha }_{V}({\lambda }_{{\rm{L}}})}.$$

Transformation (Eq. 21) also holds for the classical case when we exchange the Talbot coefficients ${B}_{n}^{({\rm{k}})}(\chi )$ in Eq. (22) with classical coefficients ${C}_{n}^{({\rm{k}})}(\chi )$ the effect of the grating on classical ballistic trajectories. These coefficients are not periodic in *χ*. This changes $\sin (\pi \chi )\to \pi \chi$ and $\cos (\pi \chi )\to 1$ in Eq. (23)^44,45^. For *χ* → 0, ${B}_{n}^{({\rm{k}})}(0)$ and ${C}_{n}^{({\rm{k}})}(0)$ are identical and describe the behavior of a purely absorptive grating. Both classical and quantum coefficients then simplify in terms of the modified Bessel functions *I*_n_(*x*):

25 $${B}_{n}^{({\rm{k}})}(0)={(-1)}^{n}\ \exp \left(-\frac{{n}_{0}^{({\rm{k}})}}{2}\right){I}_{n}\left(\frac{{n}_{0}^{({\rm{k}})}}{2}\right).$$

This formalism allows to describe the beam propagation through a Talbot-Lau-Interferometer as sequences of free propagation followed by transmission through a grating. The initial state at the first grating is assumed to be an incoherent mixture with a spatial extension *X*_0_ ≫ *d* and a momentum *P*_0_ ≫ *h*∕*d*. The initial Wigner function at the first grating *w*_0_(*x*, *p*_*x*_) is described by the transverse momentum distribution *D*(*p*_*x*_), which is gained by integration of the three dimensional momentum density distribution *μ*(*p*_*x*_, *p*_*y*_, *p*_*z*_) over two dimensions: *D*(*p*_*x*_) = ∫*dp*_*y*_ *dp*_*z*_*μ*(*p*_*x*_, *p*_*y*_, *p*_*z*_). This leads to

26 $${w}_{0}\left(x,{p}_{x}\right)=\frac{1}{{X}_{0}}D({p}_{x}+{p}_{\gamma }),$$

where *p*_*γ*_ denotes the additional constant momentum due to the tilt. According to Eq. (21), transmission through the first grating with the transmission kernel *T*^(1)^ leads to ${w}_{1}\left(x,{p}_{x}\right)$. Note that *p*_*γ*_ is a constant momentum. Therefore the substitution in the integral $\int dp^{\prime} {T}^{(1)}(x,{p}_{x}-p^{\prime} )D(p^{\prime} )$ with $p^{\prime} ={p}_{0}+{p}_{\gamma }$ leads to $dp^{\prime} =d({p}_{0}+{p}_{\gamma })=d{p}_{0}$. With iterative usage of Eq. (13) and Eq. (21), the Wigner function transforms to *w*_2_(*x*, *p*_*x*_) after free propagation for a time *t* = *T*_1_, then to *w*_3_(*x*, *p*_*x*_) after transmission through the second grating and after another free propagation for a time *t* = *T*_2_, it transforms to *w*_4_(*x*, *p*_*x*_), which denotes the state of the beam before interacting with the third grating. The corresponding transformations are listed below:

27 $${w}_{1}\left(x,{p}_{x}\right)=	\frac{1}{{X}_{0}}\int d{p}_{0}{T}^{(1)}\left(x,{p}_{x}-{p}_{0}-{p}_{\gamma }\right)D\left({p}_{0}+{p}_{\gamma }\right)\\ {w}_{2}\left(x,{p}_{x}\right)=	\frac{1}{{X}_{0}}\int d{p}_{0}{T}^{(1)}\left(x-\frac{{p}_{x}\ {T}_{1}}{m}+\frac{{p}_{\gamma }{T}_{1}}{m}+\frac{g\ {T}_{1}^{2}}{2},{p}_{x}-{p}_{0}-{p}_{\gamma }-a\ m\ {T}_{1}\right)D\left({p}_{0}+{p}_{\gamma }\right),\\ {w}_{3}\left(x,{p}_{x}\right)=	\frac{1}{{X}_{0}}\int d{p}_{1}{T}^{(2)}\left(x,{p}_{x}-{p}_{1}\right)\int d{p}_{0}{T}^{(1)}\left(x-\frac{{p}_{1}\ {T}_{1}}{m}+\frac{{p}_{\gamma }{T}_{1}}{m}+\frac{g\ {T}_{1}^{2}}{2},{p}_{1}-{p}_{0}-{p}_{\gamma }-a\ m\ {T}_{1}\right)D\left({p}_{0}+{p}_{\gamma }\right),\\ {w}_{4}\left(x,{p}_{x}\right)=	\frac{1}{{X}_{0}}\int d{p}_{1}{T}^{(2)}\left(x-\frac{{p}_{x}\ {T}_{2}}{m}+\frac{{p}_{\gamma }{T}_{2}}{m}+\frac{g\ {T}_{2}^{2}}{2},{p}_{x}-{p}_{1}-{p}_{\gamma }-a\ m\ {T}_{2}\right) \\ \quad\qquad\qquad 	\times {\int} d{p}_{0}\ {T}^{(1)}\left(x-\frac{{p}_{x}\ {T}_{2}}{m}-\frac{{p}_{1}\ {T}_{1}}{m}+\frac{{p}_{\gamma }\left({T}_{1}+{T}_{2}\right)}{m}+\frac{g\left({T}_{1}^{2}+{T}_{2}^{2}\right)}{2},{p}_{1}-{p}_{0}-{p}_{\gamma }-a\ m\ {T}_{1}\right)D\left({p}_{0}+{p}_{\gamma }\right).$$

The third grating masks the fringe pattern of the traversing molecular beam in space. Finally, all molecules are detected independent of their transverse momentum. Therefore only the spatial density distribution of the beam is needed which is calculated by integrating *w*_4_(*x*, *p*_*x*_) over the momentum. $\widetilde{D}(x)$ is the Fourier transform of the momentum distribution^44,46^

28 $$\widetilde{D}\left(x\right)=\int d{p}_{x}\ {e}^{-i{p}_{x}x/\hslash }D\left({p}_{x}\right).$$

Due to the broad initial momentum, $\widetilde{D}(x)$ is assumed to be very narrow and to peak around $\widetilde{D}(0)=1$. So only index pairs (*k*, *l*) which fulfill $\left|k{T}_{1}+l{T}_{2}\right|\ll {T}_{{\rm{T}}}$ contribute to the integral of ${w}_{4}\left(x,{p}_{x}\right)$ in Eq. (27). In the near-resonant and symmetric approximation one assumes *T*_1_ = *T* and *T*_2_ = *T* + *τ*, where *τ* denotes a small delay of the grating timing compared to the Talbot time $\left|\tau \right|\ll {T}_{{\rm{T}}}$. This restricts the index pairs (*k*, *l*) to *k* = −*l* and changes the Wigner function *w*_4_(*x*) to

29 $${w}_{4}\left(x\right)=\frac{1}{{X}_{0}}\sum _{l}\widetilde{D}\left(\frac{l\tau }{{T}_{{\rm{T}}}}d\right){B}_{-l}^{(1)}\left(\frac{l\tau }{{T}_{{\rm{T}}}}d\right) \times {B}_{2l}^{(2)}\left(\frac{l\left(T+\tau \right)}{{T}_{{\rm{T}}}}\right)\exp \left[\frac{2\pi il}{d}\left(\Delta x\right)\right],$$

30 $$\Delta x=\Delta {x}_{{\rm{s}}}-\frac{{p}_{\gamma }\tau }{m}-g{T}^{2}-2g\tau T-\frac{g{\tau }^{2}}{2}.$$

Here *Δx*_s_ denotes the relative grating shift *Δx*_s_ = *Δx*_1_ − 2*Δx*_2_ + *Δx*_3_. The spatial distribution of *w*_4_(*x*) is scanned using the third grating, which acts as a purely absorptive mask. Therefore one can use ${B}_{-l}^{(3)}(0)$ from Eq. (25). For sufficiently small delays *τ* and due to the random phase of the impinging matter-wave, *G*^(1)^ can also be treated as a purely absorptive grating, with ${B}_{-l}^{(1)}(d\ l\tau /{T}_{{\rm{T}}})={B}_{-l}^{(1)}(0)$.

Convolution of Eq. (29) with the transmission Kernel *T*^(3)^(*x*, *p*) and integration over the whole phase space leads to the detected signal *S*(*Δx*) behind the third grating

31 $$S\left(\Delta x\right)=\sum _{l}{S}_{l}\exp \left[\frac{2\pi il}{d}\Delta x(\Delta {x}_{{\rm{s}}},T,\tau )\right],$$

32 $${S}_{l}=\widetilde{D}\left(\frac{l\tau }{{T}_{{\rm{T}}}}d\right){B}_{-l}^{(1)}\left(0\right){B}_{2l}^{(2)}\left(\frac{l\left(T+\tau \right)}{{T}_{{\rm{T}}}}\right){B}_{-l}^{(3)}\left(0\right).$$

The periodic modulation of $S\left(\Delta x\right)$ is observed by scanning over the phase of Eq. (31). This can be done either by changing the grating shift *Δx*_s_ or the momentum contribution *p*_*γ*_*τ*∕*m*. A slight delay of the third grating timing *τ* modulates the phase, as well as the signal amplitude, due to the contribution of *τ* to the sharp peaked function $\widetilde{D}$.

### Periodicity of the observed fringes

**Fig. 5:**
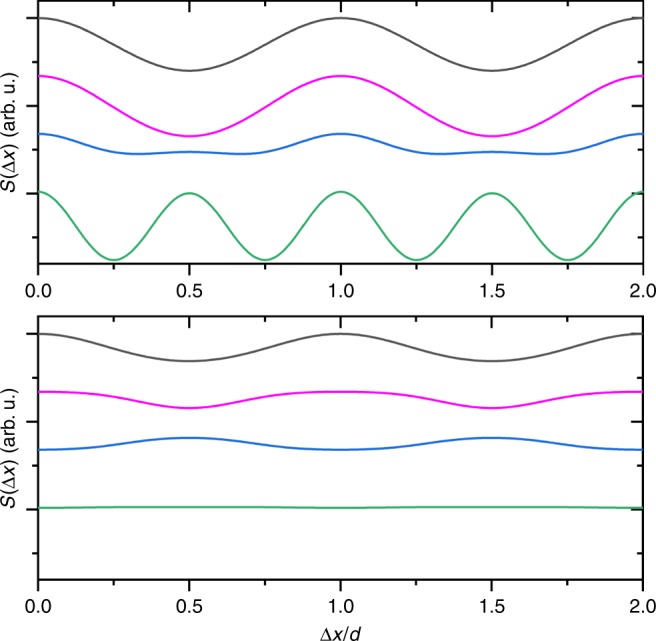
Expected periodicity of the fringes: Molecular density patterns at *G*^(3)^ in the *n* = 1/2 Talbot order, as a function of the grating strength, i.e., the mean number of photons absorbed in the grating antinode: *n*_0,eff_ = 3 (black), 4 (magenta), 6 (blue), and 12 (green). The top and bottom panel show the quantum and classical expectation, respectively.

The simulations in Fig. 3 show that only quantum interference can explain the observed normalized fringe signal and the role of complex (real and imaginary) optical gratings is important. In an interferometer with three binary transmission gratings, quantum theory would predict a halving of the fringe period at half the Talbot order *n* = 1/2^47^. However, this prediction becomes intensity dependent when using optical gratings. Figure 5 shows the quantum and classical expectation for the molecular density at *G*^(3)^ in the half Talbot order as a function of laser power, i.e., as a function of the mean number of photons absorbed in the antinodes of each grating, *n*_0,eff_. For *n*_0,eff_ = 3, as used in our experiments, *d*-periodic fringes are expected in both the *n* = 1 and the *n* = 1/2 Talbot order, classically and quantum mechanically. However, for *n* = 1, the *quantitative* distinction between both models is clear and with a large margin in our experiment (see main text).

## Data Availability

The data that support the findings of this study—in particular the raw data of Fig. 3 incl. measured data, error bars, fit, classical and quantum prediction—are available from the corresponding authors upon reasonable request.

## Code availability

The proprietary Matlab code that has been developed throughout the years and used to produce the classical and quantum predictions as described in the methods section is available to the editor and reviewers on request from the corresponding authors.

## References

[1] Cronin AD, Schmiedmayer J, Pritchard DE (2009). Optics and interferometry with atoms and molecules. Rev. Mod. Phys..

[2] Tino, G. & Kasevich, M. *Atom Interferometry*, Vol. 188. In *Proceedings of the International School of Physics “Enrico Fermi”* (IOS, Varenna, 2014).

[3] Kovachy T (2015). Quantum superposition at the half-metre scale. Nature.

[4] Schlippert D (2014). uantum test of the universality of free fall. Phys. Rev. Lett..

[5] Hamilton P (2015). Atom-interferometry constraints on dark energy. Science.

[6] Asenbaum P (2017). Phase shift in an atom interferometer due to spacetime curvature across its wave function. Phys. Rev. Lett..

[7] Rosi G (2017). Quantum test of the equivalence principle for atoms in coherent superposition of internal energy states. Nat. Commun..

[8] Geiger R (2011). Detecting inertial effects with airborne matter-wave interferometry. Nat. Commun..

[9] Savoie D (2018). Interleaved atom interferometry for high-sensitivity inertial measurements. Sci. Adv..

[10] Parker RH, Yu C, Zhong W, Estey B, Müller H (2018). Measurement of the fine-structure constant as a test of the standard model. Science.

[11] Arndt M (1999). Wave-particle duality of c60 molecules. Nature.

[12] Haslinger P (2013). A universal matter-wave interferometer with optical ionization gratings in the time domain. Nat. Phys..

[13] Fein YY (2019). Quantum superposition of molecules beyond 25 kDa. Nat.Phys..

[14] Gring M (2010). Influence of conformational molecular dynamics on matter wave interferometry. Phys. Rev. A.

[15] Eibenberger S, Cheng X, Cotter JP, Arndt M (2014). Absolute absorption cross sections from photon recoil in a matter-wave interferometer. Phys. Rev. Lett..

[16] Rodewald J (2017). New avenues for matter-wave-enhanced spectroscopy. Appl. Phys. B.

[17] Jarrold MF (2000). Peptides and proteins in the vapor phase. Annu. Rev. Phys. Chem..

[18] Mairhofer L (2017). Quantum-assisted metrology of neutral vitamins in the gas phase. Angew. Chem. Int. Ed..

[19] Schätti J (2017). Tailoring the volatility and stability of oligopeptides. J. Mass. Spectrom..

[20] Karas DM, Bahr DU, Ingendoh A, Hillenkamp PDF (1989). Laser desorption/ionization lass spectrometry of proteins of mass 100,000 to 250,000 dalton. Angew. Chem. Int. Ed..

[21] Fenn JB, Mann M, Meng CK, Wong SF, Whitehouse CM (1989). Electrospray ionization for mass spectrometry of large biomolecules. Science.

[22] Marksteiner M, Haslinger P, Sclafani M, Arndt M (2009). Uv and vuv ionization of organic molecules, clusters, and complexes. J. Phys. Chem. A.

[23] Knochenmuss R (2006). Ion formation mechanisms in uv-maldi. Analyst.

[24] Schätti J (2018). Pushing the mass limit for intact launch and photoionization of large neutral biopolymers. Commun. Chem..

[25] Gahlmann ST, A. Park, Zewail AH (2009). Structure of isolated biomolecules by electron diffraction-laser desorption: Uracil and guanine. J. Am. Chem. Soc..

[26] Rodewald J (2018). Isotope-selective high-order interferometry with large organic molecules in free fall. New J. Phys..

[27] Nimmrichter S, Haslinger P, Hornberger K, Arndt M (2011). Concept of an ionizing time-domain matter-wave interferometer. New J. Phys..

[28] Dörre N, Haslinger P, Rodewald J, Geyer P, Arndt M (2015). Refined model for talbot-lau matter-wave optics with pulsed photodepletion gratings. J. Opt. Soc. Am..

[29] Krylov AI, Gill PM (2013). Q-chem: an engine for innovation. Wiley Interdiscip. Rev. Comput. Mol. Sci..

[30] Rohrdanz MA, Martins KM, Herbert JM (2009). A long-range-corrected density functional that performs well for both ground-state properties and time-dependent density functional theory excitation energies, including charge-transfer excited states. J. Chem. Phys..

[31] Wu T, Kalugina YN, Thakkar AJ (2015). Choosing a density functional for static molecular polarizabilities. Chem. Phys. Lett..

[32] Kussmann J, Ochsenfeld C (2007). A density matrix-based method for the linear-scaling calculation of dynamic second- and third-order properties at the hartree-fock and kohn-sham density functional theory levels. J. Chem. Phys..

[33] Arndt M (2014). De Broglie’s meter stick: making measurements with matter waves. Phys. Today.

[34] Becke AD (1988). Density-functional exchange-energy approximation with correct asymptotic behavior. Phys. Rev. A.

[35] Lee C, Yang W, Parr RG (1988). Development of the colle-salvetti correlation-energy formula into a functional of the electron density. Phys. Rev. B.

[36] Titov AV, Ufimtsev IS, Luehr N, Martinez TJ (2013). Generating efficient quantum chemistry codes for novel architectures. J. Chem. Theory Comput..

[37] Ufimtsev IS, Martinez TJ (2009). Quantum chemistry on graphical processing units. 3. analytical energy gradients, geometry optimization, and first principles molecular dynamics. J. Chem. Theory Comput..

[38] Bussi G, Parrinello M (2007). Accurate sampling using langevin dynamics. Phys. Rev. E.

[39] Wu T, Kalugina YN, Thakkar AJ (2015). Choosing a density functional for static molecular polarizabilities. Chem. Phys. Lett..

[40] Nimmrichter S, Hornberger K (2008). Theory of Talbot-Lau interference beyond the eikonal approximation. Phys. Rev. A.

[41] Wigner E (1932). On the quantum correction for thermodynamic equilibrium. Phys. Rev..

[42] Hornberger K, Sipe JE, Arndt M (2004). Theory of decoherence in a matter wave talbot-lau interferometer. Phys. Rev. A.

[43] Rodewald, J. Experiments With a Pulsed Talbot Lau Matter-wave Interferometer. PhD Thesis, University of Vienna (2017).

[44] Nimmrichter S, Hornberger K (2013). Macroscopicity of mechanical quantum superposition states. Phys. Rev. Lett..

[45] Nimmrichter S, Hornberger K, Haslinger P, Arndt M (2011). Testing spontaneous localization theories with matter-wave interferometry. Phys. Rev. A.

[46] Nimmrichter, S. Matter Wave Talbot-Lau Interferometry Beyond the Eikonal Approximation. PhD Thesis, University of Vienna (2007).

[47] Clauser, J. F. & Li, S. Generalized Talbot-Lau atom interferometry. (ed. Berman, P. R.) In *Atom Interferometry* 121–151 (Academic Press, San Diego, 1997).

